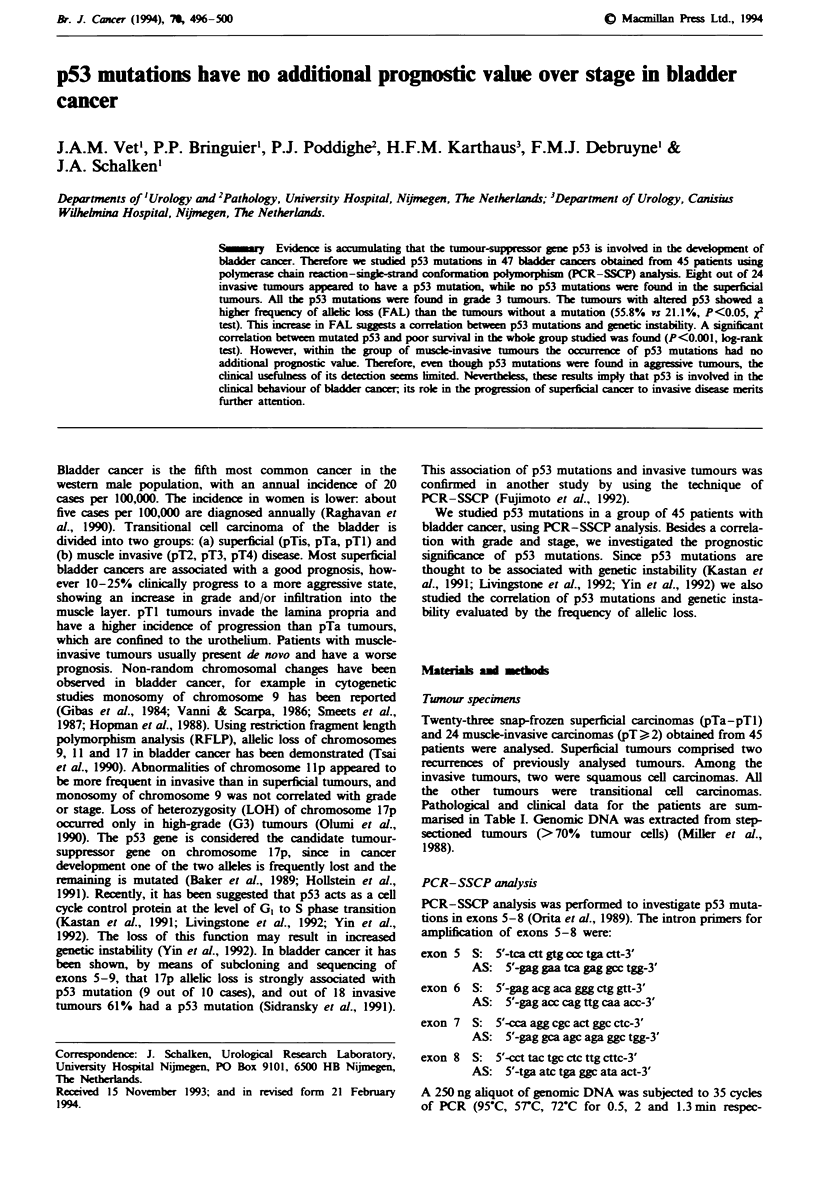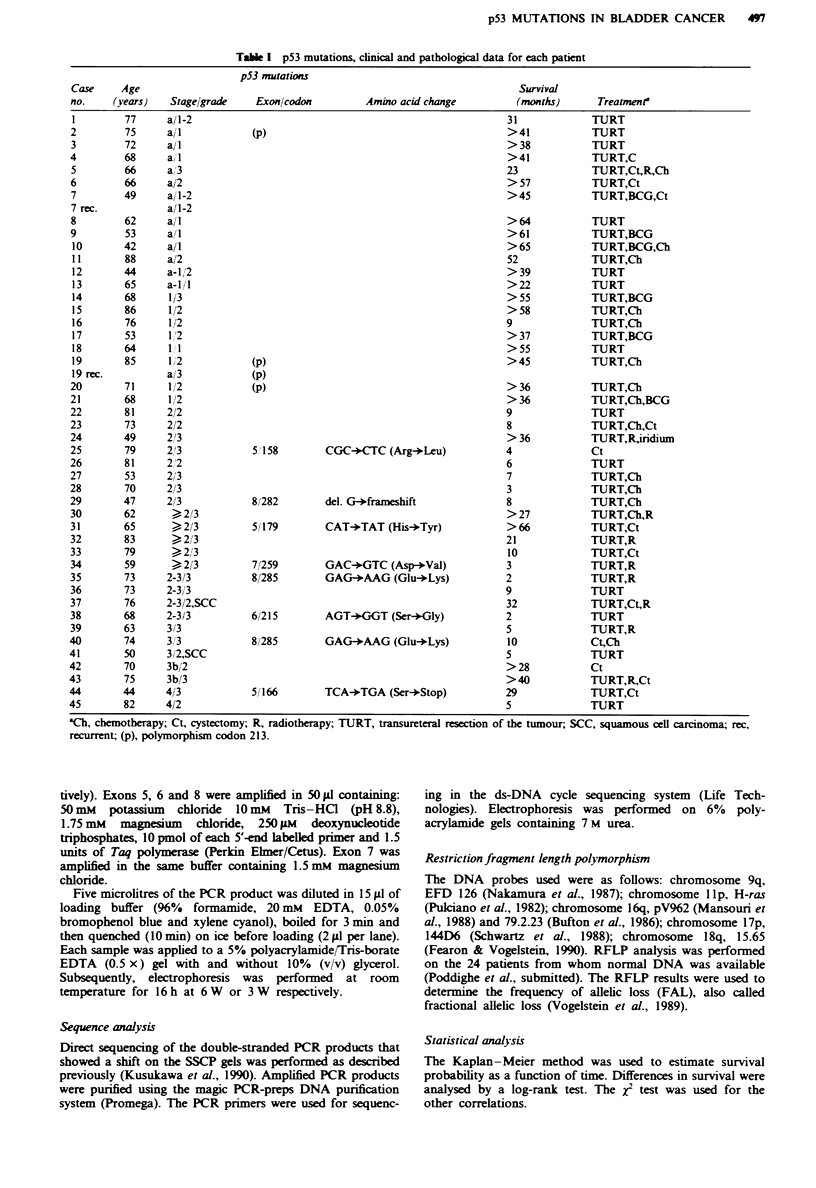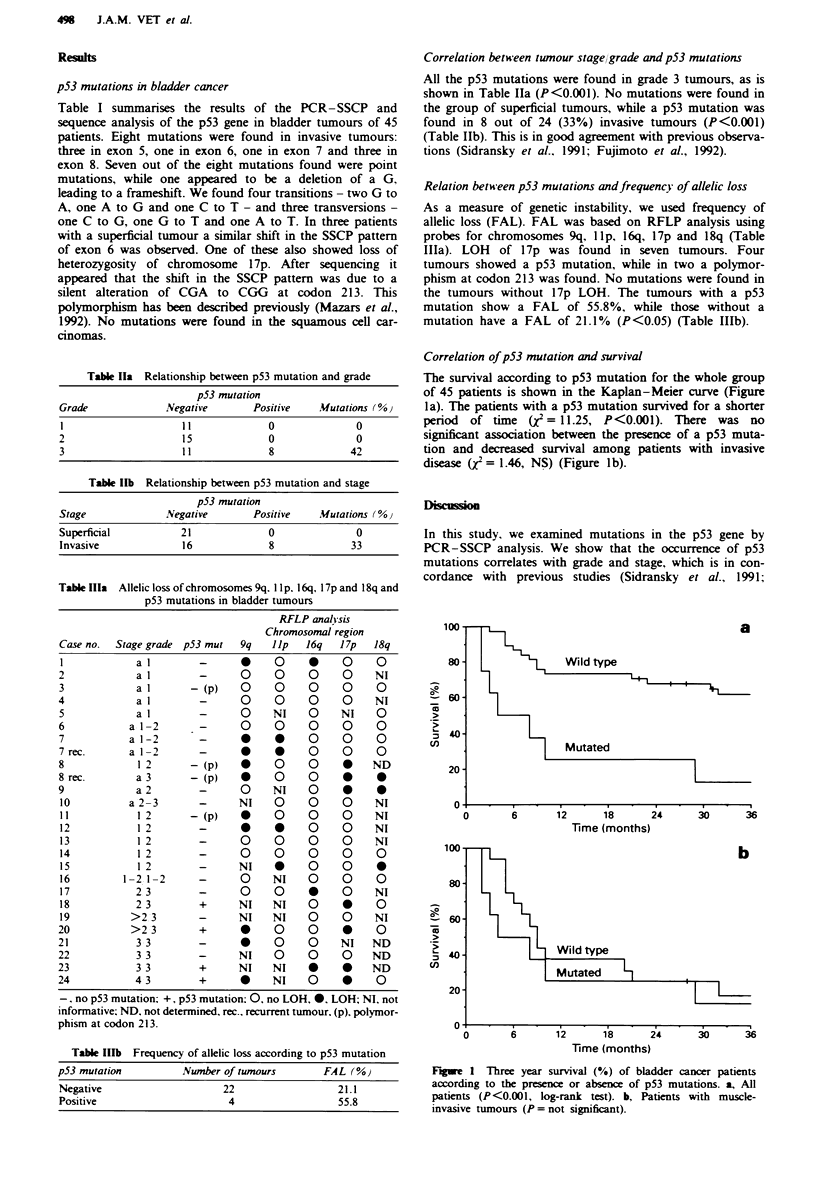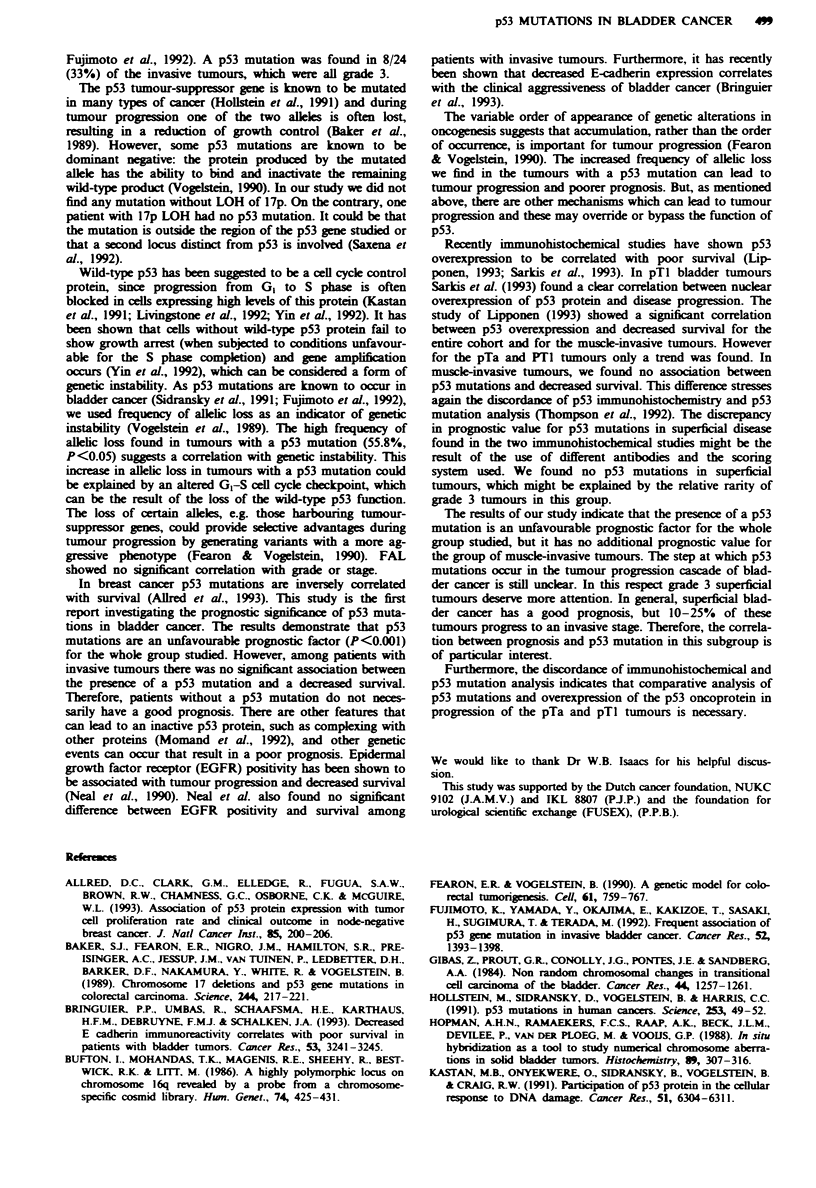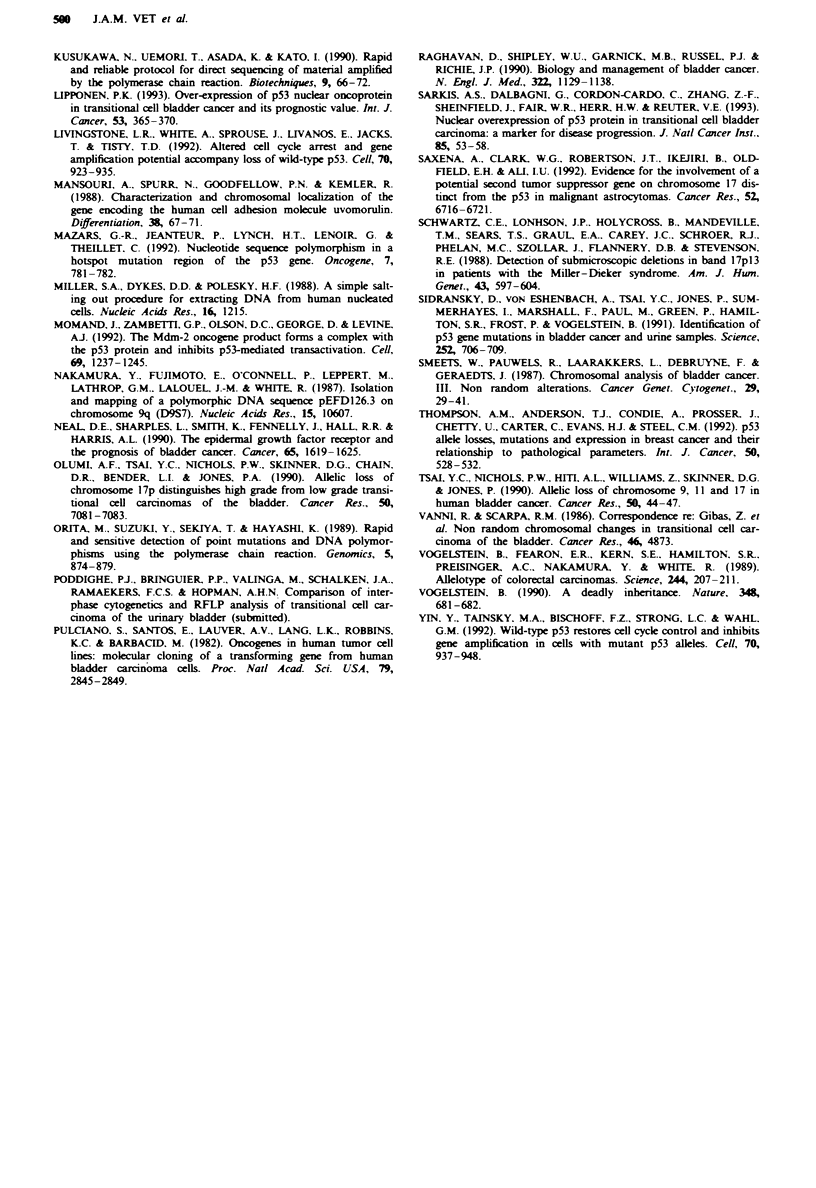# p53 mutations have no additional prognostic value over stage in bladder cancer.

**DOI:** 10.1038/bjc.1994.334

**Published:** 1994-09

**Authors:** J. A. Vet, P. P. Bringuier, P. J. Poddighe, H. F. Karthaus, F. M. Debruyne, J. A. Schalken

**Affiliations:** Department of Urology, University Hospital, Nijmegen, The Netherlands.

## Abstract

Evidence is accumulating that the tumour-suppressor gene p53 is involved in the development of bladder cancer. Therefore we studied p53 mutations in 47 bladder cancers obtained from 45 patients using polymerase chain reaction-single-strand conformation polymorphism (PCR-SSCP) analysis. Eight out of 24 invasive tumours appeared to have a p53 mutation, while no p53 mutations were found in the superficial tumours. All the p53 mutations were found in grade 3 tumours. The tumours with altered p53 showed a higher frequency of allelic loss (FAL) than the tumours without a mutation (55.8% vs 21.1%, P < 0.05, chi 2 test). This increase in FAL suggests a correlation between p53 mutations and genetic instability. A significant correlation between mutated p53 and poor survival in the whole group studied was found (P < 0.001, log-rank test). However, within the group of muscle-invasive tumours the occurrence of p53 mutations had no additional prognostic value. Therefore, even though p53 mutations were found in aggressive tumours, the clinical usefulness of its detection seems limited. Nevertheless, these results imply that p53 is involved in the clinical behaviour of bladder cancer; its role in the progression of superficial cancer to invasive disease merits further attention.


Br. J. Cancer (1994), 70, 496-500 © Macmillan Press Ltd., 1994

### p53 mutations have no additional prognostic value over stage in bladder cancer

J.A.M. Vet[1], P.P. Bringuier[1], P.J. Poddighe[2], H.F.M. Karthaus[3], F.M.J. Debruyne[1] & J.A. Schalken[1]

*Departments of [1]Urology and [2]Pathology, University Hospital, Nijmegen, The Netherlands; [3]Department of Urology, Canisius Wilhelmina Hospital, Nijmegen, The Netherlands.*

**Summary** Evidence is accumulating that the tumour-suppressor gene p53 is involved in the development of bladder cancer. Therefore we studied p53 mutations in 47 bladder cancers obtained from 45 patients using polymerase chain reaction-single-strand conformation polymorphism (PCR-SSCP) analysis. Eight out of 24 invasive tumours appeared to have a p53 mutation, while no p53 mutations were found in the superficial tumours. All the p53 mutations were found in grade 3 tumours. The tumours with altered p53 showed a higher frequency of allelic loss (FAL) than the tumours without a mutation (55.8% *vs* 21.1%, $P<0.05$, $\chi^2$ test). This increase in FAL suggests a correlation between p53 mutations and genetic instability. A significant correlation between mutated p53 and poor survival in the whole group studied was found ($P<0.001$, log-rank test). However, within the group of muscle-invasive tumours the occurrence of p53 mutations had no additional prognostic value. Therefore, even though p53 mutations were found in aggressive tumours, the clinical usefulness of its detection seems limited. Nevertheless, these results imply that p53 is involved in the clinical behaviour of bladder cancer; its role in the progression of superficial cancer to invasive disease merits further attention.

Bladder cancer is the fifth most common cancer in the western male population, with an annual incidence of 20 cases per 100,000. The incidence in women is lower: about five cases per 100,000 are diagnosed annually (Raghavan *et al.*, 1990). Transitional cell carcinoma of the bladder is divided into two groups: (a) superficial (pTis, pTa, pT1) and (b) muscle invasive (pT2, pT3, pT4) disease. Most superficial bladder cancers are associated with a good prognosis, however 10-25% clinically progress to a more aggressive state, showing an increase in grade and/or infiltration into the muscle layer. pT1 tumours invade the lamina propria and have a higher incidence of progression than pTa tumours, which are confined to the urothelium. Patients with muscle-invasive tumours usually present *de novo* and have a worse prognosis. Non-random chromosomal changes have been observed in bladder cancer, for example in cytogenetic studies monosomy of chromosome 9 has been reported (Gibas *et al.*, 1984; Vanni & Scarpa, 1986; Smeets *et al.*, 1987; Hopman *et al.*, 1988). Using restriction fragment length polymorphism analysis (RFLP), allelic loss of chromosomes 9, 11 and 17 in bladder cancer has been demonstrated (Tsai *et al.*, 1990). Abnormalities of chromosome 11p appeared to be more frequent in invasive than in superficial tumours, and monosomy of chromosome 9 was not correlated with grade or stage. Loss of heterozygosity (LOH) of chromosome 17p occurred only in high-grade (G3) tumours (Olumi *et al.*, 1990). The p53 gene is considered the candidate tumour-suppressor gene on chromosome 17p, since in cancer development one of the two alleles is frequently lost and the remaining is mutated (Baker *et al.*, 1989; Hollstein *et al.*, 1991). Recently, it has been suggested that p53 acts as a cell cycle control protein at the level of $G_1$ to S phase transition (Kastan *et al.*, 1991; Livingstone *et al.*, 1992; Yin *et al.*, 1992). The loss of this function may result in increased genetic instability (Yin *et al.*, 1992). In bladder cancer it has been shown, by means of subcloning and sequencing of exons 5-9, that 17p allelic loss is strongly associated with p53 mutation (9 out of 10 cases), and out of 18 invasive tumours 61% had a p53 mutation (Sidransky *et al.*, 1991). This association of p53 mutations and invasive tumours was confirmed in another study by using the technique of PCR-SSCP (Fujimoto *et al.*, 1992).

We studied p53 mutations in a group of 45 patients with bladder cancer, using PCR-SSCP analysis. Besides a correlation with grade and stage, we investigated the prognostic significance of p53 mutations. Since p53 mutations are thought to be associated with genetic instability (Kastan *et al.*, 1991; Livingstone *et al.*, 1992; Yin *et al.*, 1992) we also studied the correlation of p53 mutations and genetic instability evaluated by the frequency of allelic loss.

Correspondence: J. Schalken, Urological Research Laboratory, University Hospital Nijmegen, PO Box 9101, 6500 HB Nijmegen, The Netherlands.
Received 15 November 1993; and in revised form 21 February 1994.

### Materials and methods

### *Tumour specimens*

Twenty-three snap-frozen superficial carcinomas (pTa-pT1) and 24 muscle-invasive carcinomas (pT$\geqslant$2) obtained from 45 patients were analysed. Superficial tumours comprised two recurrences of previously analysed tumours. Among the invasive tumours, two were squamous cell carcinomas. All the other tumours were transitional cell carcinomas. Pathological and clinical data for the patients are summarised in Table I. Genomic DNA was extracted from step-sectioned tumours (>70% tumour cells) (Miller *et al.*, 1988).

### *PCR-SSCP analysis*

PCR-SSCP analysis was performed to investigate p53 mutations in exons 5-8 (Orita *et al.*, 1989). The intron primers for amplification of exons 5-8 were:

exon 5 S: 5′-tca ctt gtg ccc tga ctt-3′
AS: 5′-gag gaa tca gag gcc tgg-3′

exon 6 S: 5′-gag acg aca ggg ctg gtt-3′
AS: 5′-gag acc cag ttg caa acc-3′

exon 7 S: 5′-cca agg cgc act ggc ctc-3′
AS: 5′-gag gca agc aga ggc tgg-3′

exon 8 S: 5′-cct tac tgc ctc ttg cttc-3′
AS: 5′-tga atc tga ggc ata act-3′

A 250 ng aliquot of genomic DNA was subjected to 35 cycles of PCR (95°C, 57°C, 72°C for 0.5, 2 and 1.3 min respec-

**Table I** p53 mutations, clinical and pathological data for each patient

| Case no. | Age (years) | Stage/grade | p53 mutations Exon/codon | Amino acid change | Survival (months) | Treatment[a] |
|---|---|---|---|---|---|---|
| 1 | 77 | a/1-2 | | | 31 | TURT |
| 2 | 75 | a/1 | (p) | | >41 | TURT |
| 3 | 72 | a/1 | | | >38 | TURT |
| 4 | 68 | a/1 | | | >41 | TURT,C |
| 5 | 66 | a/3 | | | 23 | TURT,Ct,R,Ch |
| 6 | 66 | a/2 | | | >57 | TURT,Ct |
| 7 | 49 | a/1-2 | | | >45 | TURT,BCG,Ct |
| 7 rec. | | a/1-2 | | | | |
| 8 | 62 | a/1 | | | >64 | TURT |
| 9 | 53 | a/1 | | | >61 | TURT,BCG |
| 10 | 42 | a/1 | | | >65 | TURT,BCG,Ch |
| 11 | 88 | a/2 | | | 52 | TURT,Ch |
| 12 | 44 | a-1/2 | | | >39 | TURT |
| 13 | 65 | a-1/1 | | | >22 | TURT |
| 14 | 68 | 1/3 | | | >55 | TURT,BCG |
| 15 | 86 | 1/2 | | | >58 | TURT,Ch |
| 16 | 76 | 1/2 | | | 9 | TURT,Ch |
| 17 | 53 | 1/2 | | | >37 | TURT,BCG |
| 18 | 64 | 1/1 | | | >55 | TURT |
| 19 | 85 | 1/2 | (p) | | >45 | TURT,Ch |
| 19 rec. | | a/3 | (p) | | | |
| 20 | 71 | 1/2 | (p) | | >36 | TURT,Ch |
| 21 | 68 | 1/2 | | | >36 | TURT,Ch,BCG |
| 22 | 81 | 2/2 | | | 9 | TURT |
| 23 | 73 | 2/2 | | | 8 | TURT,Ch,Ct |
| 24 | 49 | 2/3 | | | >36 | TURT,R,iridium |
| 25 | 79 | 2/3 | 5/158 | CGC→CTC (Arg→Leu) | 4 | Ct |
| 26 | 81 | 2/2 | | | 6 | TURT |
| 27 | 53 | 2/3 | | | 7 | TURT,Ch |
| 28 | 70 | 2/3 | | | 3 | TURT,Ch |
| 29 | 47 | 2/3 | 8/282 | del. G→frameshift | 8 | TURT,Ch |
| 30 | 62 | ≥2/3 | | | >27 | TURT,Ch,R |
| 31 | 65 | ≥2/3 | 5/179 | CAT→TAT (His→Tyr) | >66 | TURT,Ct |
| 32 | 83 | ≥2/3 | | | 21 | TURT,R |
| 33 | 79 | ≥2/3 | | | 10 | TURT,Ct |
| 34 | 59 | ≥2/3 | 7/259 | GAC→GTC (Asp→Val) | 3 | TURT,R |
| 35 | 73 | 2-3/3 | 8/285 | GAG→AAG (Glu→Lys) | 2 | TURT,R |
| 36 | 73 | 2-3/3 | | | 9 | TURT |
| 37 | 76 | 2-3/2,SCC | | | 32 | TURT,Ct,R |
| 38 | 68 | 2-3/3 | 6/215 | AGT→GGT (Ser→Gly) | 2 | TURT |
| 39 | 63 | 3/3 | | | 5 | TURT,R |
| 40 | 74 | 3/3 | 8/285 | GAG→AAG (Glu→Lys) | 10 | Ct,Ch |
| 41 | 50 | 3/2,SCC | | | 5 | TURT |
| 42 | 70 | 3b/2 | | | >28 | Ct |
| 43 | 75 | 3b/3 | | | >40 | TURT,R,Ct |
| 44 | 44 | 4/3 | 5/166 | TCA→TGA (Ser→Stop) | 29 | TURT,Ct |
| 45 | 82 | 4/2 | | | 5 | TURT |

[a]Ch, chemotherapy; Ct, cystectomy; R, radiotherapy; TURT, transureteral resection of the tumour; SCC, squamous cell carcinoma; rec, recurrent; (p), polymorphism codon 213.

tively). Exons 5, 6 and 8 were amplified in 50 µl containing: 50 mM potassium chloride 10 mM Tris-HCl (pH 8.8), 1.75 mM magnesium chloride, 250 µM deoxynucleotide triphosphates, 10 pmol of each 5′-end labelled primer and 1.5 units of *Taq* polymerase (Perkin Elmer/Cetus). Exon 7 was amplified in the same buffer containing 1.5 mM magnesium chloride.

Five microlitres of the PCR product was diluted in 15 µl of loading buffer (96% formamide, 20 mM EDTA, 0.05% bromophenol blue and xylene cyanol), boiled for 3 min and then quenched (10 min) on ice before loading (2 µl per lane). Each sample was applied to a 5% polyacrylamide/Tris-borate EDTA (0.5 ×) gel with and without 10% (v/v) glycerol. Subsequently, electrophoresis was performed at room temperature for 16 h at 6 W or 3 W respectively.

*Sequence analysis*

Direct sequencing of the double-stranded PCR products that showed a shift on the SSCP gels was performed as described previously (Kusukawa *et al.*, 1990). Amplified PCR products were purified using the magic PCR-preps DNA purification system (Promega). The PCR primers were used for sequencing in the ds-DNA cycle sequencing system (Life Technologies). Electrophoresis was performed on 6% polyacrylamide gels containing 7 M urea.

*Restriction fragment length polymorphism*

The DNA probes used were as follows: chromosome 9q, EFD 126 (Nakamura *et al.*, 1987); chromosome 11p, H-*ras* (Pulciano *et al.*, 1982); chromosome 16q, pV962 (Mansouri *et al.*, 1988) and 79.2.23 (Bufton *et al.*, 1986); chromosome 17p, 144D6 (Schwartz *et al.*, 1988); chromosome 18q, 15.65 (Fearon & Vogelstein, 1990). RFLP analysis was performed on the 24 patients from whom normal DNA was available (Poddighe *et al.*, submitted). The RFLP results were used to determine the frequency of allelic loss (FAL), also called fractional allelic loss (Vogelstein *et al.*, 1989).

*Statistical analysis*

The Kaplan-Meier method was used to estimate survival probability as a function of time. Differences in survival were analysed by a log-rank test. The $\chi^2$ test was used for the other correlations.

### Results

### *p53 mutations in bladder cancer*

Table I summarises the results of the PCR-SSCP and sequence analysis of the p53 gene in bladder tumours of 45 patients. Eight mutations were found in invasive tumours: three in exon 5, one in exon 6, one in exon 7 and three in exon 8. Seven out of the eight mutations found were point mutations, while one appeared to be a deletion of a G, leading to a frameshift. We found four transitions – two G to A, one A to G and one C to T – and three transversions – one C to G, one G to T and one A to T. In three patients with a superficial tumour a similar shift in the SSCP pattern of exon 6 was observed. One of these also showed loss of heterozygosity of chromosome 17p. After sequencing it appeared that the shift in the SSCP pattern was due to a silent alteration of CGA to CGG at codon 213. This polymorphism has been described previously (Mazars *et al.*, 1992). No mutations were found in the squamous cell carcinomas.

**Table IIa** Relationship between p53 mutation and grade

| Grade | p53 mutation Negative | Positive | Mutations (%) |
|---|---|---|---|
| 1 | 11 | 0 | 0 |
| 2 | 15 | 0 | 0 |
| 3 | 11 | 8 | 42 |

**Table IIb** Relationship between p53 mutation and stage

| Stage | p53 mutation Negative | Positive | Mutations (%) |
|---|---|---|---|
| Superficial | 21 | 0 | 0 |
| Invasive | 16 | 8 | 33 |

**Table IIIa** Allelic loss of chromosomes 9q, 11p, 16q, 17p and 18q and p53 mutations in bladder tumours

| | | | RFLP analysis Chromosomal region | | | | |
|---|---|---|---|---|---|---|---|
| *Case no.* | *Stage/grade* | *p53 mut* | *9q* | *11p* | *16q* | *17p* | *18q* |
| 1 | a/1 | – | ● | ○ | ● | ○ | ○ |
| 2 | a/1 | – | ○ | ○ | ○ | ○ | NI |
| 3 | a/1 | – (p) | ○ | ○ | ○ | ○ | ○ |
| 4 | a/1 | – | ○ | ○ | ○ | ○ | NI |
| 5 | a/1 | – | ○ | NI | ○ | NI | ○ |
| 6 | a/1–2 | – | ○ | ○ | ○ | ○ | ○ |
| 7 | a/1–2 | – | ● | ● | ○ | ○ | ○ |
| 7 rec. | a/1–2 | – | ● | ● | ○ | ○ | ○ |
| 8 | 1/2 | – (p) | ● | ○ | ○ | ● | ND |
| 8 rec. | a/3 | – (p) | ● | ○ | ○ | ● | ● |
| 9 | a/2 | – | ○ | NI | ○ | ● | ● |
| 10 | a/2–3 | – | NI | ○ | ○ | ○ | NI |
| 11 | 1/2 | – (p) | ● | ○ | ○ | ○ | NI |
| 12 | 1/2 | – | ● | ● | ○ | ○ | NI |
| 13 | 1/2 | – | ○ | ○ | ○ | ○ | NI |
| 14 | 1/2 | – | ○ | ○ | ○ | ○ | ○ |
| 15 | 1/2 | – | NI | ● | ○ | ○ | ● |
| 16 | 1–2/1–2 | – | ○ | NI | ○ | ○ | ○ |
| 17 | 2/3 | – | ○ | ○ | ● | ○ | NI |
| 18 | 2/3 | + | NI | NI | ○ | ● | ○ |
| 19 | >2/3 | – | NI | NI | ○ | ○ | NI |
| 20 | >2/3 | + | ● | ○ | ○ | ● | ○ |
| 21 | 3/3 | – | ● | ○ | ○ | NI | ND |
| 22 | 3/3 | – | NI | ○ | ○ | ○ | ND |
| 23 | 3/3 | + | NI | NI | ● | ● | ND |
| 24 | 4/3 | + | ● | NI | ○ | ● | ○ |

–, no p53 mutation; +, p53 mutation; ○, no LOH, ●, LOH; NI, not informative; ND, not determined, rec., recurrent tumour, (p), polymorphism at codon 213.

**Table IIIb** Frequency of allelic loss according to p53 mutation

| *p53 mutation* | *Number of tumours* | *FAL (%)* |
|---|---|---|
| Negative | 22 | 21.1 |
| Positive | 4 | 55.8 |

### *Correlation between tumour stage/grade and p53 mutations*

All the p53 mutations were found in grade 3 tumours, as is shown in Table IIa ($P<0.001$). No mutations were found in the group of superficial tumours, while a p53 mutation was found in 8 out of 24 (33%) invasive tumours ($P<0.001$) (Table IIb). This is in good agreement with previous observations (Sidransky *et al.*, 1991; Fujimoto *et al.*, 1992).

### *Relation between p53 mutations and frequency of allelic loss*

As a measure of genetic instability, we used frequency of allelic loss (FAL). FAL was based on RFLP analysis using probes for chromosomes 9q, 11p, 16q, 17p and 18q (Table IIIa). LOH of 17p was found in seven tumours. Four tumours showed a p53 mutation, while in two a polymorphism at codon 213 was found. No mutations were found in the tumours without 17p LOH. The tumours with a p53 mutation show a FAL of 55.8%, while those without a mutation have a FAL of 21.1% ($P<0.05$) (Table IIIb).

### *Correlation of p53 mutation and survival*

The survival according to p53 mutation for the whole group of 45 patients is shown in the Kaplan-Meier curve (Figure 1a). The patients with a p53 mutation survived for a shorter period of time ($\chi^2 = 11.25$, $P<0.001$). There was no significant association between the presence of a p53 mutation and decreased survival among patients with invasive disease ($\chi^2 = 1.46$, NS) (Figure 1b).

### Discussion

In this study, we examined mutations in the p53 gene by PCR-SSCP analysis. We show that the occurrence of p53 mutations correlates with grade and stage, which is in concordance with previous studies (Sidransky *et al.*, 1991;

**Figure 1** Three year survival (%) of bladder cancer patients according to the presence or absence of p53 mutations. **a**, All patients ($P<0.001$, log-rank test). **b**, Patients with muscle-invasive tumours ($P$ = not significant).

Fujimoto *et al.*, 1992). A p53 mutation was found in 8/24 (33%) of the invasive tumours, which were all grade 3.

The p53 tumour-suppressor gene is known to be mutated in many types of cancer (Hollstein *et al.*, 1991) and during tumour progression one of the two alleles is often lost, resulting in a reduction of growth control (Baker *et al.*, 1989). However, some p53 mutations are known to be dominant negative: the protein produced by the mutated allele has the ability to bind and inactivate the remaining wild-type product (Vogelstein, 1990). In our study we did not find any mutation without LOH of 17p. On the contrary, one patient with 17p LOH had no p53 mutation. It could be that the mutation is outside the region of the p53 gene studied or that a second locus distinct from p53 is involved (Saxena *et al.*, 1992).

Wild-type p53 has been suggested to be a cell cycle control protein, since progression from $G_1$ to S phase is often blocked in cells expressing high levels of this protein (Kastan *et al.*, 1991; Livingstone *et al.*, 1992; Yin *et al.*, 1992). It has been shown that cells without wild-type p53 protein fail to show growth arrest (when subjected to conditions unfavourable for the S phase completion) and gene amplification occurs (Yin *et al.*, 1992), which can be considered a form of genetic instability. As p53 mutations are known to occur in bladder cancer (Sidransky *et al.*, 1991; Fujimoto *et al.*, 1992), we used frequency of allelic loss as an indicator of genetic instability (Vogelstein *et al.*, 1989). The high frequency of allelic loss found in tumours with a p53 mutation (55.8%, $P<0.05$) suggests a correlation with genetic instability. This increase in allelic loss in tumours with a p53 mutation could be explained by an altered $G_1$–S cell cycle checkpoint, which can be the result of the loss of the wild-type p53 function. The loss of certain alleles, e.g. those harbouring tumour-suppressor genes, could provide selective advantages during tumour progression by generating variants with a more aggressive phenotype (Fearon & Vogelstein, 1990). FAL showed no significant correlation with grade or stage.

In breast cancer p53 mutations are inversely correlated with survival (Allred *et al.*, 1993). This study is the first report investigating the prognostic significance of p53 mutations in bladder cancer. The results demonstrate that p53 mutations are an unfavourable prognostic factor ($P<0.001$) for the whole group studied. However, among patients with invasive tumours there was no significant association between the presence of a p53 mutation and a decreased survival. Therefore, patients without a p53 mutation do not necessarily have a good prognosis. There are other features that can lead to an inactive p53 protein, such as complexing with other proteins (Momand *et al.*, 1992), and other genetic events can occur that result in a poor prognosis. Epidermal growth factor receptor (EGFR) positivity has been shown to be associated with tumour progression and decreased survival (Neal *et al.*, 1990). Neal *et al.* also found no significant difference between EGFR positivity and survival among patients with invasive tumours. Furthermore, it has recently been shown that decreased E-cadherin expression correlates with the clinical aggressiveness of bladder cancer (Bringuier *et al.*, 1993).

The variable order of appearance of genetic alterations in oncogenesis suggests that accumulation, rather than the order of occurrence, is important for tumour progression (Fearon & Vogelstein, 1990). The increased frequency of allelic loss we find in the tumours with a p53 mutation can lead to tumour progression and poorer prognosis. But, as mentioned above, there are other mechanisms which can lead to tumour progression and these may override or bypass the function of p53.

Recently immunohistochemical studies have shown p53 overexpression to be correlated with poor survival (Lipponen, 1993; Sarkis *et al.*, 1993). In pT1 bladder tumours Sarkis *et al.* (1993) found a clear correlation between nuclear overexpression of p53 protein and disease progression. The study of Lipponen (1993) showed a significant correlation between p53 overexpression and decreased survival for the entire cohort and for the muscle-invasive tumours. However for the pTa and PT1 tumours only a trend was found. In muscle-invasive tumours, we found no association between p53 mutations and decreased survival. This difference stresses again the discordance of p53 immunohistochemistry and p53 mutation analysis (Thompson *et al.*, 1992). The discrepancy in prognostic value for p53 mutations in superficial disease found in the two immunohistochemical studies might be the result of the use of different antibodies and the scoring system used. We found no p53 mutations in superficial tumours, which might be explained by the relative rarity of grade 3 tumours in this group.

The results of our study indicate that the presence of a p53 mutation is an unfavourable prognostic factor for the whole group studied, but it has no additional prognostic value for the group of muscle-invasive tumours. The step at which p53 mutations occur in the tumour progression cascade of bladder cancer is still unclear. In this respect grade 3 superficial tumours deserve more attention. In general, superficial bladder cancer has a good prognosis, but 10–25% of these tumours progress to an invasive stage. Therefore, the correlation between prognosis and p53 mutation in this subgroup is of particular interest.

Furthermore, the discordance of immunohistochemical and p53 mutation analysis indicates that comparative analysis of p53 mutations and overexpression of the p53 oncoprotein in progression of the pTa and pT1 tumours is necessary.

We would like to thank Dr W.B. Isaacs for his helpful discussion.

This study was supported by the Dutch cancer foundation, NUKC 9102 (J.A.M.V.) and IKL 8807 (P.J.P.) and the foundation for urological scientific exchange (FUSEX), (P.P.B.).

### References

ALLRED, D.C., CLARK, G.M., ELLEDGE, R., FUGUA, S.A.W., BROWN, R.W., CHAMNESS, G.C., OSBORNE, C.K. & MCGUIRE, W.L. (1993). Association of p53 protein expression with tumor cell proliferation rate and clinical outcome in node-negative breast cancer. *J. Natl Cancer Inst.*, **85**, 200–206.

BAKER, S.J., FEARON, E.R., NIGRO, J.M., HAMILTON, S.R., PREISINGER, A.C., JESSUP, J.M., VAN TUINEN, P., LEDBETTER, D.H., BARKER, D.F., NAKAMURA, Y., WHITE, R. & VOGELSTEIN, B. (1989). Chromosome 17 deletions and p53 gene mutations in colorectal carcinoma. *Science*, **244**, 217–221.

BRINGUIER, P.P., UMBAS, R., SCHAAFSMA, H.E., KARTHAUS, H.F.M., DEBRUYNE, F.M.J. & SCHALKEN, J.A. (1993). Decreased E cadherin immunoreactivity correlates with poor survival in patients with bladder tumors. *Cancer Res.*, **53**, 3241–3245.

BUFTON, I., MOHANDAS, T.K., MAGENIS, R.E., SHEEHY, R., BESTWICK, R.K. & LITT, M. (1986). A highly polymorphic locus on chromosome 16q revealed by a probe from a chromosome-specific cosmid library. *Hum. Genet.*, **74**, 425–431.

FEARON, E.R. & VOGELSTEIN, B. (1990). A genetic model for colorectal tumorigenesis. *Cell*, **61**, 759–767.

FUJIMOTO, K., YAMADA, Y., OKAJIMA, E., KAKIZOE, T., SASAKI, H., SUGIMURA, T. & TERADA, M. (1992). Frequent association of p53 gene mutation in invasive bladder cancer. *Cancer Res.*, **52**, 1393–1398.

GIBAS, Z., PROUT, G.R., CONOLLY, J.G., PONTES, J.E. & SANDBERG, A.A. (1984). Non random chromosomal changes in transitional cell carcinoma of the bladder. *Cancer Res.*, **44**, 1257–1261.

HOLLSTEIN, M., SIDRANSKY, D., VOGELSTEIN, B. & HARRIS, C.C. (1991). p53 mutations in human cancers. *Science*, **253**, 49–52.

HOPMAN, A.H.N., RAMAEKERS, F.C.S., RAAP, A.K., BECK, J.L.M., DEVILEE, P., VAN DER PLOEG, M. & VOOIJS, G.P. (1988). *In situ* hybridization as a tool to study numerical chromosome aberrations in solid bladder tumors. *Histochemistry*, **89**, 307–316.

KASTAN, M.B., ONYEKWERE, O., SIDRANSKY, B., VOGELSTEIN, B. & CRAIG, R.W. (1991). Participation of p53 protein in the cellular response to DNA damage. *Cancer Res.*, **51**, 6304–6311.

KUSUKAWA, N., UEMORI, T., ASADA, K. & KATO, I. (1990). Rapid and reliable protocol for direct sequencing of material amplified by the polymerase chain reaction. *Biotechniques*, **9**, 66-72.

LIPPONEN, P.K. (1993). Over-expression of p53 nuclear oncoprotein in transitional cell bladder cancer and its prognostic value. *Int. J. Cancer*, **53**, 365-370.

LIVINGSTONE, L.R., WHITE, A., SPROUSE, J., LIVANOS, E., JACKS, T. & TISTY, T.D. (1992). Altered cell cycle arrest and gene amplification potential accompany loss of wild-type p53. *Cell*, **70**, 923-935.

MANSOURI, A., SPURR, N., GOODFELLOW, P.N. & KEMLER, R. (1988). Characterization and chromosomal localization of the gene encoding the human cell adhesion molecule uvomorulin. *Differentiation*, **38**, 67-71.

MAZARS, G.-R., JEANTEUR, P., LYNCH, H.T., LENOIR, G. & THEILLET, C. (1992). Nucleotide sequence polymorphism in a hotspot mutation region of the p53 gene. *Oncogene*, **7**, 781-782.

MILLER, S.A., DYKES, D.D. & POLESKY, H.F. (1988). A simple salting out procedure for extracting DNA from human nucleated cells. *Nucleic Acids Res.*, **16**, 1215.

MOMAND, J., ZAMBETTI, G.P., OLSON, D.C., GEORGE, D. & LEVINE, A.J. (1992). The Mdm-2 oncogene product forms a complex with the p53 protein and inhibits p53-mediated transactivation. *Cell*, **69**, 1237-1245.

NAKAMURA, Y., FUJIMOTO, E., O'CONNELL, P., LEPPERT, M., LATHROP, G.M., LALOUEL, J.-M. & WHITE, R. (1987). Isolation and mapping of a polymorphic DNA sequence pEFD126.3 on chromosome 9q (D9S7). *Nucleic Acids Res.*, **15**, 10607.

NEAL, D.E., SHARPLES, L., SMITH, K., FENNELLY, J., HALL, R.R. & HARRIS, A.L. (1990). The epidermal growth factor receptor and the prognosis of bladder cancer. *Cancer*, **65**, 1619-1625.

OLUMI, A.F., TSAI, Y.C., NICHOLS, P.W., SKINNER, D.G., CHAIN, D.R., BENDER, L.I. & JONES, P.A. (1990). Allelic loss of chromosome 17p distinguishes high grade from low grade transitional cell carcinomas of the bladder. *Cancer Res.*, **50**, 7081-7083.

ORITA, M., SUZUKI, Y., SEKIYA, T. & HAYASHI, K. (1989). Rapid and sensitive detection of point mutations and DNA polymorphisms using the polymerase chain reaction. *Genomics*, **5**, 874-879.

PODDIGHE, P.J., BRINGUIER, P.P., VALINGA, M., SCHALKEN, J.A., RAMAEKERS, F.C.S. & HOPMAN, A.H.N. Comparison of interphase cytogenetics and RFLP analysis of transitional cell carcinoma of the urinary bladder (submitted).

PULCIANO, S., SANTOS, E., LAUVER, A.V., LANG, L.K., ROBBINS, K.C. & BARBACID, M. (1982). Oncogenes in human tumor cell lines: molecular cloning of a transforming gene from human bladder carcinoma cells. *Proc. Natl Acad. Sci. USA*, **79**, 2845-2849.

RAGHAVAN, D., SHIPLEY, W.U., GARNICK, M.B., RUSSEL, P.J. & RICHIE, J.P. (1990). Biology and management of bladder cancer. *N. Engl. J. Med.*, **322**, 1129-1138.

SARKIS, A.S., DALBAGNI, G., CORDON-CARDO, C., ZHANG, Z.-F., SHEINFIELD, J., FAIR, W.R., HERR, H.W. & REUTER, V.E. (1993). Nuclear overexpression of p53 protein in transitional cell bladder carcinoma: a marker for disease progression. *J. Natl Cancer Inst.*, **85**, 53-58.

SAXENA, A., CLARK, W.G., ROBERTSON, J.T., IKEJIRI, B., OLDFIELD, E.H. & ALI, I.U. (1992). Evidence for the involvement of a potential second tumor suppressor gene on chromosome 17 distinct from the p53 in malignant astrocytomas. *Cancer Res.*, **52**, 6716-6721.

SCHWARTZ, C.E., LONHSON, J.P., HOLYCROSS, B., MANDEVILLE, T.M., SEARS, T.S., GRAUL, E.A., CAREY, J.C., SCHROER, R.J., PHELAN, M.C., SZOLLAR, J., FLANNERY, D.B. & STEVENSON, R.E. (1988). Detection of submicroscopic deletions in band 17p13 in patients with the Miller-Dieker syndrome. *Am. J. Hum. Genet.*, **43**, 597-604.

SIDRANSKY, D., VON ESHENBACH, A., TSAI, Y.C., JONES, P., SUMMERHAYES, I., MARSHALL, F., PAUL, M., GREEN, P., HAMILTON, S.R., FROST, P. & VOGELSTEIN, B. (1991). Identification of p53 gene mutations in bladder cancer and urine samples. *Science*, **252**, 706-709.

SMEETS, W., PAUWELS, R., LAARAKKERS, L., DEBRUYNE, F. & GERAEDTS, J. (1987). Chromosomal analysis of bladder cancer. III. Non random alterations. *Cancer Genet. Cytogenet.*, **29**, 29-41.

THOMPSON, A.M., ANDERSON, T.J., CONDIE, A., PROSSER, J., CHETTY, U., CARTER, C., EVANS, H.J. & STEEL, C.M. (1992). p53 allele losses, mutations and expression in breast cancer and their relationship to pathological parameters. *Int. J. Cancer*, **50**, 528-532.

TSAI, Y.C., NICHOLS, P.W., HITI, A.L., WILLIAMS, Z., SKINNER, D.G. & JONES, P. (1990). Allelic loss of chromosome 9, 11 and 17 in human bladder cancer. *Cancer Res.*, **50**, 44-47.

VANNI, R. & SCARPA, R.M. (1986). Correspondence re: Gibas, Z. *et al.* Non random chromosomal changes in transitional cell carcinoma of the bladder. *Cancer Res.*, **46**, 4873.

VOGELSTEIN, B., FEARON, E.R., KERN, S.E., HAMILTON, S.R., PREISINGER, A.C., NAKAMURA, Y. & WHITE, R. (1989). Allelotype of colorectal carcinomas. *Science*, **244**, 207-211.

VOGELSTEIN, B. (1990). A deadly inheritance. *Nature*, **348**, 681-682.

YIN, Y., TAINSKY, M.A., BISCHOFF, F.Z., STRONG, L.C. & WAHL, G.M. (1992). Wild-type p53 restores cell cycle control and inhibits gene amplification in cells with mutant p53 alleles. *Cell*, **70**, 937-948.